# A comparative study on the clinical effect of the treatment of pathological scars in young and middle-aged women with the combination of Chinese and western medicine

**DOI:** 10.1097/MD.0000000000020623

**Published:** 2020-06-19

**Authors:** Ya-hong Liu, Jun Xiang, Pei-pei Han, Chun Yang, Yu-zhen Wang, Wei Wang, Ping-an Zhang

**Affiliations:** The Second Affiliated Hospital of Shaanxi University of Chinese Medicine, Xianyang, Shaanxi, P.R. China.

**Keywords:** clinical trials, pathological scars, traditional Chinese medicine

## Abstract

**Introduction:**

Pathological scar is the abnormal manifestation of skin fiber hyperplasia caused by the failure of normal healing after skin damage. At present, there are many clinical treatments for pathological scars. However, there is no cure for clinically effective pathological scars with high recurrence rate. In this study, we will use a combination of Chinese and western medicine treatment methods to evaluate the clinical efficacy and related indicators of young and middle-aged female patients who meet pathological scars, looking for an objective and effective treatment method for pathological scars.

**Methods/design:**

In this study, we will use our own front-to-back clinical research method. We plan to include 120 young and middle-aged female patients who meet the diagnostic criteria for pathological scars. The untreated pathological scars of the enrolled patients will be used as blank controls. The intervention group will be given conventional western medicine treatment and combined Chinese and western medicine treatment. The assessment of scar area, color, hardness, thickness, itching, and pain was recorded for 8 weeks of treatment.

**Discussion:**

This trial may provide evidence regarding the clinical effectiveness, safety, and cost-effectiveness of traditional Chinese medicine for patients with pathological scars.

**Trial registration:**

ClinicalTrials.gov, ChiCTR2000032187, Registered on April 22, 2020.

## 1 Introduction

Pathological scars are the abnormal manifestation of skin fiber hyperplasia caused by the failure of normal healing after skin damage.^[1]^ At present, there are many clinical treatments for pathological scars. Clinically, it is mostly treated by surgery, hormone therapy, radiotherapy, and laser, but there is no cure for clinically effective pathological scars with high recurrence rate.^[2]^ Traditional Chinese medicine (TCM) has a unique understanding of pathological scars. TCM holds that the pathological scars is due to the deficiency of positive Qi after skin trauma, and the evil poison is stagnant on the surface of the skin, which gradually causes the phlegm and congestion of the body to form on the surface, thereby forming pathological scars. And it is related to their own constitution.^[3,4]^ Some Chinese medical experts hold that this is related to long-term dampness in the body or dampness of the lungs and stomach, coupled with skin trauma, stagnation of blood and blood stasis, and eventually pathological scars are formed. Therefore, in the treatment of pathological scars, TCM usually starts with the methods of promoting blood circulation and removing blood stasis, orally combined with external treatment, or supplemented with acupuncture and point injection. TCM has a good clinical effect on the treatment of this disease. At present, the pathogenesis of pathological scars is still unclear, but the theories supported by more scholars include genetics, vascular endothelial dysfunction, inflammation, and immunology.^[5]^ Pathological scars is mainly characterized by excessive inflammation. Modern medicine believes that pathological scars are related to the body's immune response.^[6,7]^ On the one hand, the inflammatory response stimulates the immune response, and the cytokines produced increase the inflammatory response. In contrast, the immune response also affects the inflammatory response. TCM plays a regulatory role in the body's systemic or local inflammatory and immune responses.

TCM is currently effective in preventing and treating pathological scars and has unique advantages. Oral administration of TCM, external application of TCM, acupuncture and moxibustion, and combination of traditional Chinese and western medicine can be selected. Through the analysis of syndrome differentiation of TCM, according to the actual situation of the patient, choose the appropriate prescription medicine to improve the patient's Qi stagnation, blood stasis and meridian obstruction pathogenesis as a whole, and then achieve satisfactory results. The mechanism of TCM treatment of pathological scars is mainly to inhibit the proliferation of fibroblasts and the synthesis of collagen. At present, we should fully integrate Chinese medicine pharmacology. Strengthen the research on the treatment of pathological scars with TCM, and screen out the drugs with the exact curative effect and less side effects to inhibit the scar hyperplasia. While giving full play to the advantages of TCM treatment, we should also draw on the advantages of Western medicine treatment and carry out integrated Chinese and western medicine treatment to improve clinical efficacy and reduce the recurrence rate. Although the curative effect of TCM on pathological scars is certain, there is a problem that the standard of curative effect is not uniform. Through the consensus of the Chinese medicine industry, the release of industry guidelines for the treatment of pathological scars by Chinese medicine, and the uniform evaluation of efficacy standards, are of great significance for the study of Chinese medicine treatment of pathological scars. Therefore, in this study, we will use our own front-to-back clinical research methods to evaluate the clinical efficacy and related indicators of young and middle-aged female patients who meet pathological scars, and to find an objective and effective treatment method for pathological scars.

## 2 Methods/design

### 2.1 Study design and settings

**Figure 1:**
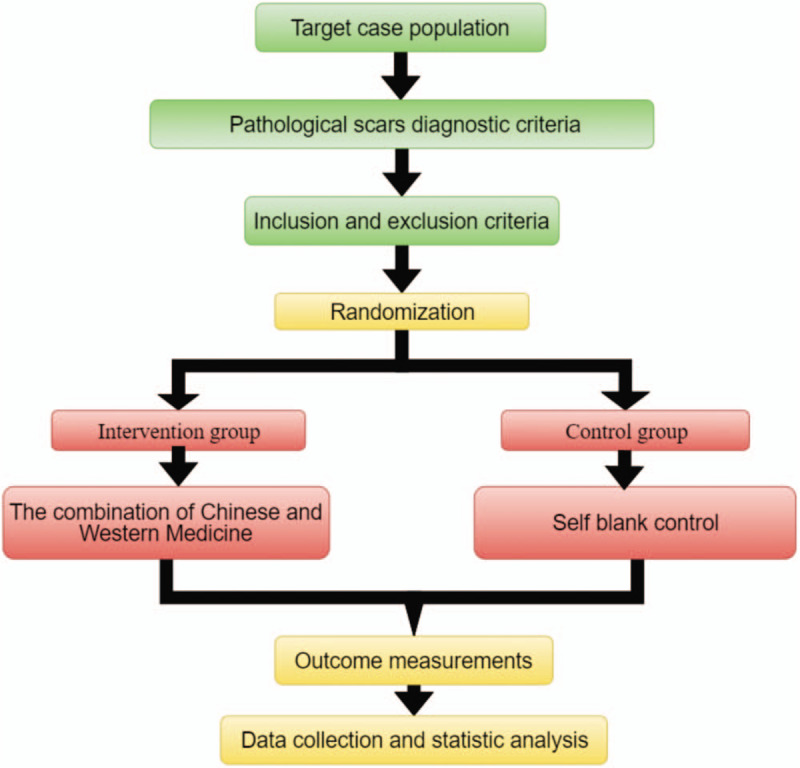
Study design flow chart.

This study will be conducted at the Second Affiliated Hospital of Shaanxi University of Chinese Medicine. This protocol was written and based on Standard Protocol Items: Recommendations for Interventional Trials guidelines the participants will be informed about the research, procedures, risks, and benefits by YHL (author of this protocol). If they agree, they will sign an informed consent form. Only those participants who read and agree to the protocol and who sign the informed consent form will take part of the study, following the schedule described in Figure 1.

### 2.2 Participants

The subjects of this study will be included in the outpatient and hospitalized patients from the Department of Plastic Surgery of the Second Affiliated Hospital of Shaanxi University of Chinese Medicine, and meet the diagnostic criteria for pathological scars. The research subjects gave informed notification and signed the “*Informed Consent for Pathological Scars Clinical Research*.”

#### 2.2.1 Diagnostic criteria

We will refer to the diagnostic criteria for pathological scars in “*Plastic Surgery*.”

1. Hypertrophic scars: scars caused by burns or trauma, surgery, grow like tumors, bulge on the skin surface, local thickening and hardening, not exceeding the original wound limit, the surface is reddish brown. Or accompanied by itching or pain is the main clinical manifestation.
2. Keloids: bright red or auburn, swollen, hard-textured tumors caused by acne, chickenpox, injection, insect bites, piercings, etc caused by minor trauma or no obvious incentives, most of which develop from papules to nodules. The main clinical manifestation is continuous expansion in all directions, accompanied by strong pain and itching.

#### 2.2.2 Inclusion criteria

This study will be conducted in China. Patients will be recruited from Plastic surgery departments of the Second Affiliated Hospital of Shaanxi University of Chinese Medicine. We will enroll participants based on the following inclusion criteria:

1. It meets the diagnostic criteria for pathological scars;
2. There are at least 3 pathological healing scars throughout the body.
3. Female patients of 16 ≤ age ≤ 40 years old;
4. The time for the formation of fatigue marks exceeds 3 months;
5. Those who have voluntarily signed the informed consent.

#### 2.2.3 Exclusion criteria

Patients will be excluded if they meet the following criteria:

1. People with allergies and allergies to experimental drugs;
2. Participants are between 16 and 40 years old;
3. Patients with renal cancer, bladder tuberculosis, urinary stones;
4. Patients with other malignant tumors;
5. Patients with blood system diseases, coagulation dysfunction, and autoimmune diseases;
6. Patients with severe cardiovascular disease, cerebrovascular disease, hematopoietic system disease, and neuropathy.

#### 2.2.4 Conditions for participants to suspend and withdraw from the clinical trial

Researchers participating in clinical trials should carefully record the reasons for the suspension of the trial and the relationship with the clinical trial. It is necessary to clearly record the unwillingness of the subjects to continue the clinical trials, put forward the reasons for withdrawing from the clinical trials, and record the evaluation indicators at the time of discontinuation in detail.

1. Those who cannot adhere to treatment;
2. Allergic reactions or serious adverse reactions during the test, the test should be suspended:
3. Those who have not been treated strictly according to the plan;
4. People who withdrew from the study on their own

### 2.3 Interventions

The participants included in the observation will be self-controlled, and each patient randomly selected 3 different scar treatment areas at the same site as observation specimens.

Control group: Take the untreated pathological scars of the participants as blank control. This group will not give any intervention.

**Figure 2:**
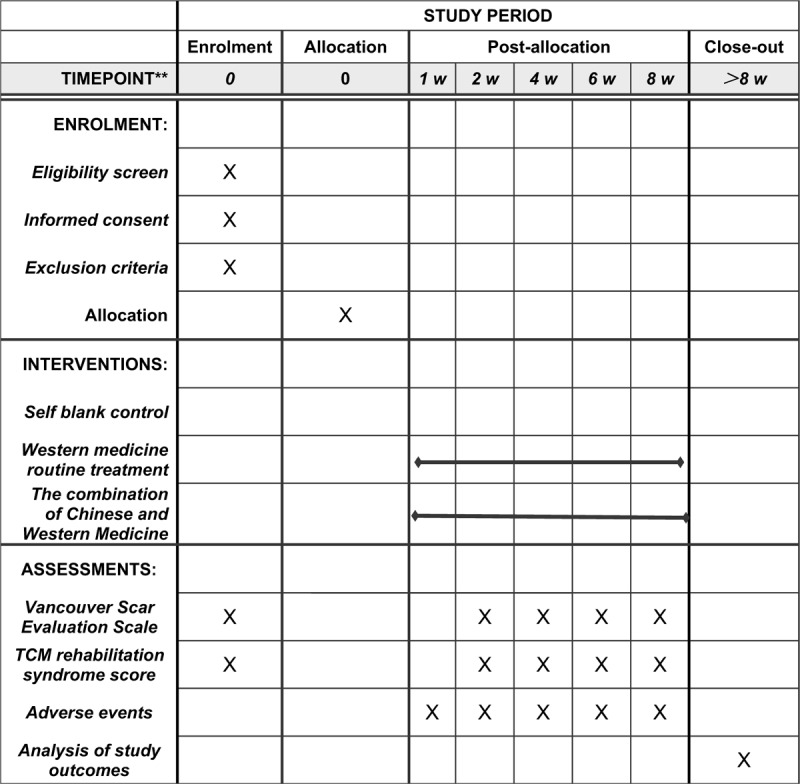
SPIRIT figure for the schedule of enrollment, interventions, and assessments. SPIRIT = Standard Protocol Items: Recommendations for Interventional Trials, TCM = traditional Chinese medicine.

Intervention group: Among the two pathological scars, group A will be treated with conventional external medicine of western medicine, and group B will be treated with oral TCM decoction combined with conventional external medicine of western medicine. The main components of the TCM prescription are: *Baizhi, Jiazhu, Leiwan, Maidong, Yuanhu, Taoren, Honghua*, and *Jingjie*. Specific treatment method: clean the scar area, apply gel to the affected area, every 3 times. TCM decoction is taken orally after meals, 3 times a day. The total application time will be 8 weeks. At the same time, closely monitor the change of the condition in order to control the deterioration of the condition in time (Fig. 2).

### 2.4 Outcome measures

The assessment of scar area, color, hardness, thickness, itching, and pain was recorded for 8 weeks of treatment. Assess fatigue marks once a week and save case photos.

#### 2.4.1 Primary outcome measures

We will use the international scar evaluation method—“*Vancouver Scar Evaluation Scale*” as the main standard for evaluating the efficacy.

1. Color: 3 points for bright red, 2 points for light red, and disappear after pressing, 1 point for non-red or some gray, 0 points for normal skin color;
2. Height of scar marks: 3 points above 9 mm, 5 mm < 2 points for height ≤9 mm, 1 point for 1 to 5 mm, 0 points for flat or slightly depressed;
3. Hardness: 3 points for hard as cartilage, 2 points for hardness like rubber, 1 point for softness, normal for softness skin score: 0 points;
4. Itching: 3 points for severe or persistent itching, 2 points for frequent and tolerable itching, sometimes 1 point for itching, and 0 points for no itching;
5. Pain: very intense 3 points for the “hyperalgesia,” 2 points for moderate-intensity allergic pain, sometimes 1 point for pain, and 0 points for no pain;
6. tightness: 3 points for strong tightness, not too tight strong and tolerable score 2 points, slight tightness score, no tightness score 0 points.

Efficacy index = (total score before treatment − total score after treatment)/total score before treatment × 100%.

#### 2.4.2 Efficacy evaluation

Refer to “*Guidelines for Clinical Research of New Chinese Medicine*.” Efficacy was evaluated by the ratio of the difference between the points before and after treatment compared to the points before treatment. Syndrome treatment efficiency = (total points before treatment total points after treatment) / total points before treatment × 100%.

1. Clinical control: clinical symptoms and signs disappeared or basically disappeared, and syndrome scores were reduced by ≥95%;
2. Significant effect: clinical symptoms and signs were significantly improved, and syndrome scores were reduced by 70%;
3. The signs and symptoms have improved, and the syndrome scores have decreased by ≥30%;
4. Ineffective: the clinical symptoms and signs have not improved significantly, and the syndrome scores have decreased by <30%.

### 2.5 Sample size calculation

The sample size for this trial is based on an expected mean difference between groups of 11 points of the Constant–Murley questionnaire, which is the minimum clinically important difference. To detect this difference between both treatments, with a value of *a* = 0.05 (probability of committing a type I error) and a statistical power of 95%, a minimum of 49 patients per group is needed. This minimal sample size estimate has been increased by 20% after considering the potential dropouts, finally including 60 patients for each group. Accordingly, the proposed experimental hypothesis is that there will be a difference of at least 11 points in the Constant–Murley questionnaire in the intervention group versus the control group. The sample size was determined using the Stata SE software, version 15.

### 2.6 Randomization and blinding

Participants will be randomly allocated to the 2 groups through a sequence of numbers generated by a computer program before starting the selection process. The group assigned to each patient will be kept in a sealed envelope with the objective of concealing the assignment to the researcher, who will decide on the entry of subjects to the study. Given the nature of the interventions, the physiotherapists, and the patients, blinding will not be possible. However, the evaluator and statistician will be blinded to which group the subjects evaluated will belong.

### 2.7 Statistical analysis

Data management uses EXCEL software to build a database, double entry, check for outstanding values, and lock. Statistical analysis will be performed using SPSS 25.0 software for statistical analysis. The normality of the measurement data is tested. The data obeying the normal distribution is Student's *t* test, which is expressed by mean ± standard deviation. The data not obeying the normal distribution is rank sum test. And marginal homogeneity test; count data are expressed by rate and composition ratio, and comparison is performed by chi-square test; repeated measurement data are expressed by mean ± standard deviation, intra-group comparison is performed by analysis of variance of repeated measurement data, and inter-group comparison is by multivariate analysis of variance (MANOVA). *P* ≤ .05 indicates that the difference is statistically significant.

### 2.8 Data management

Information obtained from the evaluation of each participant will be recorded on a paper print-out. The information will then be handwritten on a paper document case report form and entered into an Excel file for future statistical analyses. In accordance with the Personal Information Protection Act, the names of all participants will not be disclosed, and a unique identifier number given during the trial will be used to identify participants. All of the participants will be informed that the clinical data obtained in the trial will be stored in a computer and will be handled with confidentiality. The participants’ written consent will be stored by the principal investigator.

### 2.9 Ethics

The study will be conducted under the Declaration of Helsinki principles, as well as following the norms of good clinical practice. Recruitment of patients has not started in this study. The study plan will be submitted to the ethics committee of the Second Affiliated Hospital of Shaanxi University of Chinese Medicine for review. The study protocol will be approved by the ethics committee of the Second Affiliated Hospital of Shaanxi University of Chinese Medicine. The protocol of this study has been registered in the Chinese Clinical Trial Registry with the number ChiCTR2000032187.

## 3 Discussion

Scars are an inevitable result of wound healing. The collagen synthesis and metabolism of pathological scars are in an unbalanced state, which shows that collagen synthesis significantly exceeds degradation metabolism, which eventually leads to collagen accumulation.^[8]^ Through research, it was recognized that when the skin tissue was damaged and damaged to a certain depth, the early inflammatory waterfall-like reaction of the wound surface, that is, infiltration of white blood cells, macrophages, mast cells, etc.^[9]^ At the same time release a variety of cytokines, fibroblasts and myofibroblasts proliferate and synthesize a large amount of collagen and matrix, causing abnormal collagen metabolism and structural arrangement.^[10]^ Coupled with the influence of microcirculation and oxygen free radicals and other factors, it promotes the formation of pathological scars. Sometimes the granulation tissue can be completely absorbed by the surrounding normal tissues after the inflammation factor subsides without going through the scar hyperplasia process, but sometimes it continues to hyperplasia.^[11]^ Not only does it cause inconvenience to the patient's life, it also affects the mental health to a certain extent, and even affects functional exercise. The treatment of hypertrophic scars and keloids, especially keloids, is still a difficult hotspot in plastic surgery. The current common treatment methods for pathological scars include surgery, drug therapy, laser therapy, radiation therapy, compression therapy, silicone gel membrane, gene therapy, and so on.^[12]^ Among the drug treatments, western medicine treatment mainly targets extracellular matrix targeted drugs (including glucocorticoids, pyridone, collagenase, etc), cell-targeted drugs (including 5-FU, bleomycin, retinoic acid, Colchicine, etc), biological microenvironment targeting drugs (including growth factor regulating drugs, immunomodulators, anti-inflammatory drugs, anti-allergy drugs), and other directions.^[13,14]^ Western medicine has achieved obvious results in the local treatment of pathological scars, but it still cannot solve the problem of recurrence of scars. There is a certain relationship between the occurrence and development of pathological scars and the body's hyperimmune reaction.^[15]^ The understanding of pathological scars in TCM can be based on a holistic view, and the combination of Chinese medicine and western medicine is expected to explore a radical cure. TCM believes that the pathological scar is due to the deficiency of positive qi after skin trauma, and the evil poison is left on the skin surface to form a scar. And the occurrence of this disease is also related to its own constitution. TCM has definite clinical efficacy in preventing and treating pathological scars, and has unique advantages. While giving full play to the advantages of TCM, we have actively absorbed the advantages of western medicine treatment and conducted integrated Chinese and western medicine treatment. We aim to improve clinical efficacy and reduce the recurrence rate of pathological scars. Although the curative effect of TCM on pathological scars is certain, there is a problem that the standard of curative effect is not uniform. Therefore, in this study, we will use our own front-to-back clinical research methods to evaluate the clinical efficacy and related indicators of young and middle-aged female patients who meet pathological scars, and to find an objective and effective treatment method for pathological scars.

## Acknowledgments

The authors would like to thank all the trial participants. The authors are grateful for the support for this study: trial coordinating team, surgical staff, nurses, and research departments.

## Author contributions

YHL, JX, PPH, and YZW designed the study protocol and drafted the manuscript. PAZ reviewed the study protocol and drafted the manuscript. WW and CY are responsible for the statistical design and analysis as trial statistician. All authors carefully read and approved the final version of the manuscript.
